# Multi‐drug resistance and compensatory mutations in *Mycobacterium tuberculosis* in Vietnam

**DOI:** 10.1111/tmi.14104

**Published:** 2025-03-13

**Authors:** Quang Huy Nguyen, Thi Van Anh Nguyen, Anne‐Laure Bañuls

**Affiliations:** ^1^ LMI DRISA, Department of Life Sciences University of Science and Technology of Hanoi (USTH), Vietnam Academy of Science and Technology (VAST) Hanoi Vietnam; ^2^ Department of Bacteriology National Institute of Hygiene and Epidemiology (NIHE) Hanoi Vietnam; ^3^ MIVEGEC University of Montpellier, IRD, CNRS Montpellier France; ^4^ Present address: Foundation for Innovative New Diagnostics (FIND) Hanoi Vietnam

**Keywords:** Beijing, compensatory mutations, drug‐resistant mutations, fitness cost, multidrug resistance, *Mycobacterium tuberculosis*

## Abstract

**Background:**

Vietnam is a hotspot for the emergence and spread of multidrug‐resistant *Mycobacterium tuberculosis*. This study aimed to perform a retrospective study on the compensatory evolution in multidrug‐resistant *M. tuberculosis* strains and the association with drug‐resistant mutations and *M. tuberculosis* genotypes.

**Methods:**

Hundred and seventy‐three strains resistant to rifampicin (*n* = 126) and/or isoniazid (*n* = 170) (multidrug‐resistant = 123) were selected according to different drug‐resistant patterns and genotypes. The genes/promoter regions including *rpo*A, *rpo*B, *rpo*C, *kat*G, *inh*A, *inh*A promoter, *ahp*C, *ahp*C promoter, *gyr*A, *gyr*B, and *rrs* were sequenced for each strain.

**Results:**

Frequency of rifampicin‐ and isoniazid‐resistant mutations in multidrug‐resistant strains was 99.2% and 97.0%, respectively. Mutations associated with low –high levels of drug resistance with low‐ or no‐fitness costs compared to the wild type, including *rpo*B_Ser450Leu, *kat*G_Ser315Thr, *inh*A‐15(A‐T), *gyr*A_Asp94Gly, and *rrs*_A1401GA, accounted for 46.3%, 76.4%, 16.2%, 8.9%, and 11.4%, respectively, in the multidrug‐resistant strains. Beijing and Euro‐American genotype strains were associated with high‐level drug‐resistant mutations, *rpo*B_Ser450Leu, *kat*G_Ser315Thr, and *gyr*A_Asp94Gly, while East African–Indian genotype strains were associated with low to high‐level drug‐resistant mutations, *rpo*B_His445Asp, *rpo*B_His445Tyr, *inh*A‐15(C‐T) and *rrs_*A1401G. Multidrug‐resistant strains (19.5%) harboured compensatory mutations linked to rifampicin resistance in *rpo*A, *rpo*B, or *rpo*C. Notably, the frequency of compensatory mutations in Beijing genotypes was significantly higher than in East African–Indian genotypes (21.1% vs. 3.3%, OR = 7.7; 95% CI = 1.0 to 61.2, *p* = 0.03). The proportion of multidrug‐resistant strains with *rpo*B_Ser450Leu mutations carrying *rpo*A–*rpo*C mutations was higher than that of strains with other *rpo*B mutations (OR = 5.4; 95% CI = 1.4 to 21.1, *p* = 0.02) and was associated with Beijing strains. Only 1.2% (2/170) isoniazid‐resistant strains carried *aph*C‐52(C‐T) mutation in the promoter region of the *ahp*C gene, which was hypothesised to be the compensatory mutation in isoniazid‐resistant strains. Meanwhile, 11 isoniazid‐resistant strains carried a *kat*G mutation combined with either *inh*A‐8(T‐C) or *inh*A‐15(A‐T) mutations and were associated with East African–Indian strains.

**Conclusions:**

Mutations associated with high levels of drug resistance without/with low fitness costs (*rpo*B_Ser450Leu and *kat*G_Ser315Thr) along with compensatory mutations linked to rifampicin resistance were strongly associated with multidrug‐resistant *M. tuberculosis* Beijing strains in Vietnam.

## INTRODUCTION

Tuberculosis (TB) is mainly caused by *Mycobacterium tuberculosis* and is one of the deadliest infectious diseases worldwide, with 1.3 million deaths annually [1]. The emergence of drug‐resistant TB, especially multidrug‐resistant (MDR) and extensively drug‐resistant (XDR) TB, strongly threatens global TB control. Globally, an estimated 10.6 million people developed TB, in which approximately 410.000 people developed MDR/rifampicin‐resistant TB (RR‐TB) [1]. Among 2.9 million bacteriologically confirmed pulmonary TB cases, approximately 4.4% were MDR/RR‐TB, pre‐XDR, or XDR [1]. The COVID‐19 pandemic had a strong impact on TB diagnosis and treatment, and therefore the proportion of people diagnosed with MDR/RR‐TB is probably underestimated, which enhances the spread of highly drug‐resistant strains in the community.

The major mechanism of drug resistance in *M. tuberculosis* is mutation acquisition in drug resistance‐associated genes in the chromosome. In *M. tuberculosis*, the acquisition rate of resistance by spontaneous mutation is estimated to be 1 in 10^5^–10^8^ bacilli, depending on drug resistance genes and bacterial lineages [2]. Theoretically, drug‐resistant mutations often impose a fitness cost on *M. tuberculosis*, impacting key biological functions such as replication, survival, cell function, growth rate, competitive ability, virulence, or transmissibility [3]. Nevertheless, several studies have reported that some *M. tuberculosis* strains undergo low‐ or no‐cost mutations but exhibit a high level of resistance to drugs and high transmission potential [4, 5]. These findings suggest that the low‐ or no‐cost mutations enable *M. tuberculosis* strains to persist despite antibiotic pressure and do not significantly reduce the bacterium's overall fitness or ability to survive and reproduce, contributing to the continued circulation of drug‐resistant strains. Mutations with low‐ or no‐fitness cost are particularly concerning because they can facilitate the rapid spread of resistant strains. Molecular epidemiology studies revealed that MDR and XDR‐MTB strains can cause large country‐wide outbreaks similarly to drug‐susceptible strains [6, 7]. The transmission of MDR *M. tuberculosis* strains appears more common than drug‐resistance acquisition. The transmission was estimated to be approximately 96% of all MDR‐TB cases, and 61.3% of MDR‐TB in previously treated cases [8].

To overcome the fitness costs, secondary mutations can be selected that would improve or promote either the target protein itself or an alternative pathway with a similar function [9, 10]. These compensatory mutations often occur in genes encoding the same protein or in genes involved in similar metabolic pathways. These mutations mitigate the deleterious effects of the specific functions compromised by the resistance mutations. For instance, in the case of INH resistance with the absence of the *kat*G gene encoding for the KatG catalase‐peroxidase, mutations in the promoter region of the *ahp*C gene, coding for Alkyl hydroperoxide reductase C (AHPC) with a similar function to the KatG enzyme, lead to the overexpression of the *ahp*C gene to compensate for the loss of katG activity [11]. *Regarding the RIF resistance, the* mutations conferring the resistance often occur in a core region known as cluster I of the rifampicin resistance‐determining region (RRDR) of the *rpo*B gene encoding for β subunit of DNA‐dependent RNA polymerase. These mutations either decrease the transcriptional efficacy of the RpoB enzyme or alter the genome‐wide transcriptional profiles [12, 13]. Consequently, RIF‐resistance compensatory mutations can emerge in RNA polymerase subunits α (*rpo*A), β’ (*rpo*C) or even within the *rpo*B itself to alleviate the biological cost. Recent genomic studies identified several *rpo*B non‐RRDR mutations that co‐occurred with RRDR mutations in clinical isolates without *rpo*A/*rpo*C mutations and may also confer fitness cost compensation [14, 15]. Compensatory mutations are also frequently found in MDR XDR‐TB strains with resistance mutations carrying a limited fitness cost, such as rpoB_Ser450Leu [16, 17, 18]. *These mutations could, thus, play a role in the maintenance and spread of drug‐resistant strains* [10, 16, 19].

Vietnam is one of the 30 highest TB and MDR‐TB burden countries in the world. The first national TB surveillance implemented in 2006–2007 reported the TB prevalence of 307 per 100,000 population [20]. Although the TB incidence declined by an estimated 3% per year in the period between 2007 and 2017, the second national TB surveillance reported the slightly increased TB prevalence of 332 per 100.000 population, showing the TB burden in Vietnam remains high [1]. Furthermore, the country is listed among 10 nations accounted for approximately 70% of the global gap between the estimated annual global incidence of MDR/RR‐TB [1]. Only 36% of the estimated MDR/RR‐TB cases were laboratory‐confirmed and enrolled in treatment. The estimated proportion of new TB cases was about 3.6%–4% which was relatively stable in the period 2009–2022 [20, 21]. In addition, the country is facing high prevalence of *M. tuberculosis* strains with high transmissibility potential like Beijing genotypes especially in the mega‐cities [16, 18, 22, 23]. Thus, undetected or non‐controlled MDR‐TB cases could directly transmit drug‐resistant *M. tuberculosis* strains with the increased transmission fitness in the population. From a molecular point of view, little is known regarding the involvement of compensatory mechanisms in the transmission of MDR *M. tuberculosis* strains. In this context, we conducted a retrospective study on *M. tuberculosis* population collected in the framework of the second national TB prevalence survey in Vietnam in 2009 to explore the association between drug‐resistance mutations, compensatory mutations and *M. tuberculosis* lineages for better understand the circulation of highly drug‐resistant genotypes in Vietnam. Epistasis interactions between drug resistance‐conferring mutations and compensatory mutations in different genetic strains can be hypothesized.

## MATERIALS AND METHODS

### Mycobacterium tuberculosis strains

A total of 173 *M. tuberculosis* strains resistant to rifampicin, isoniazid, or both were selected from bacterial stocks of the National Institute of Hygiene and Epidemiology, Hanoi, Vietnam. These strains were originally part of the second national anti‐tuberculosis drug resistance surveillance conducted by the National Tuberculosis Control Program of Vietnam in 2009, which collected *M. tuberculosis* strains representing all regions of the country (Data S1). In this study, the strains were chosen based on their phenotypic susceptibility patterns to first‐line anti‐tuberculosis drugs (isoniazid, rifampicin, streptomycin, and ethambutol) and their classification into *M. tuberculosis* lineages, as determined by spoligotyping and 24‐loci MIRU‐VNTR profiles (Table 1 and Data S1) [24]. All observed resistance patterns, from single‐drug resistance to resistance against four drugs (quadruple resistance), were represented. For each drug‐resistance pattern, strains were selected according to their *M. tuberculosis* lineages and genotypes with criteria as follows: when a specific drug‐resistance pattern within a lineage contained fewer than 10 strains, all available strains were included in the study. Conversely, when more than 10 strains were present, at least 10 were randomly selected for analysis.

**TABLE 1:** Distribution of *M. tuberculosis* strains according to the first‐line drug resistance patterns and the genotypes.

Type	Drug resistance pattern	*M. tuberculosis* strains			
		EAI genotypes	Beijing genotypes	EA genotypes	Total
Mono resistance	H	12	11	4	27
	R	0	0	2	2
Non‐MDR	HS	3	12	4	19
	RSE	0	1	0	1
	HSE	0	1	0	1
MDR	HR	4	1	1	6
	HRS	5	11	4	20
	HRE	4	2	0	6
	HRSE	17	62	12	91
Total		45	101	27	173

*Note*: H: isoniazid, R: rifampicin, S: streptomycin, E: ethambutol; lineage 1/East African–Indian (EAI) genotypes, lineage 2/Beijing genotypes, lineage 4/Euro‐American (EA) genotypes (H, LAM, T and Unknown).

### DNA sequencing of the targeted genes

The genomic DNA extraction was performed as previously described [25]. Drug resistance‐associated genes, including *rpo*B (rifampicin resistance), *kat*G, *inh*A and its promoter (isoniazid resistance) as well as the quinolone‐resistance determining region of *gyrA* and *gyrB genes* (fluoroquinolone resistance) and rrs1400 region of rrs gene (second‐line injectable drug resistance) were amplified and sequenced. To explore the compensatory mutations of rifampicin resistance, *rpo*A, *rpo*B and *rpo*C genes were sequenced, while *ahp*C gene and its promoter region (*oxy*R‐*ahp*C) were sequenced for the detection of compensatory mutations associated with isoniazid resistance.

Each 25 μL PCR mix contained: 2.5 μL of PCR buffer 10×, 5 μL of 5× Q solution, 0.5 μL of 10 μmol primer for each (Table 2), 0.25 μL of 10 mM dNTPs, 0.2 μL of HotStarTaq DNA polymerase (Qiagen, Germany), 13 μL of H_2_O, and 3 μL of DNA supernatants. The PCR conditions were: 5 min of denaturation at 95°C, then 35 cycles (95°C for 1 min, 58–62°C for 1 min, and 72°C for 1 min [PCR length <1000 bp] and 1 min 30 seconds [PCR length >1000 bp], and 72°C for 5 min). The list of primers, sequences, and the length of PCR products used in this study are given in Table 2.

**TABLE 2:** Primers used for the DNA amplification and sequencing of genes involved in anti‐TB drug resistance and fitness‐compensatory evolution in *M. tuberculosis*.

Gene/gene promoter	Primer sequence	Annealing T^o^C	Length (bp)	Target region(s)	Reference
*rpo*B and *rpo*B promoter	F‐rpoB1: 5′‐GTCGACGCTGACCGAAGAAG‐3′	62°C	1148	Clusters I (including RRDR), II and III	25
	R‐rpoB1: 5′‐TCTCGCCGTCGTCAGTACAG‐3′				
	F‐rpoB2: 5′‐TAGTTGCGTGCGTGAGATCC‐3′	60°C	1174	Promoter and N‐terminal region	25
	R‐rpoB2: 5′‐TGGTCTGACCCTCGTGCAAG‐3′				
*kat*G	F‐katG1: 5′‐CCAACTCCTGGAAGGAATGC‐3′	58°C	1169	*kat*G315 hotspot codon and flanking coding regions	25
	R‐katG1: 5′‐AGAGGTCAGTGGCCAGCAT‐3′				
	F‐katG2: 5′‐ACGAGTGGGAGCTGACGAA‐3′	60°C	1229	remaining coding regions	25
	R‐katG2: 5′‐AACCCGAATCAGCGCACGT‐3′				
*inh*A and its promoter	F‐inhA1: 5′‐GCGACATACCTGCTGCGCAA‐3′	60°C	300	Promoter region	25
	R‐inhA2: 5′‐ATCCCCCGGTTTCCTCCGGT‐3′				
	F‐inhA3: 5′‐GACACAACACAAGGACGCA‐3′	60°C	1008	Full coding region	25
	R‐inhA4: 5′‐TGCCATTGATCGGTGATACC‐3′				
*rrs*	F‐S2: 5′‐GCGCAGATATCAGGAGG‐3′	58°C	918	1400–1500 region	25
	R‐S2: 5′‐CGCCCACTACAGACAAG‐3′				
*gyr*A *& gyr*B	F‐gyrAB: 5′‐GCAACACCGAGGTCAAATCG‐3′	62°C	1296	QRDRs of *gyr*A & *gyr*B	25
	R‐gyrAB: 5′‐CTCAGCATCTCCATCGCCAA‐3′				
*rpo*A	F‐rpoA: 5′‐AACCGATCCCAGTTCGTGAT‐3′	60°C	856	RpoA‐RpoC interaction region	This study
	R‐rpoA: 5′‐GCAGCTTGATCTTCACCTCG‐3′				
*rpo*C	F‐rpoC: 5′‐AGTCGCTTTCCGATCTGCTC‐3′	60°C	952	RpoA‐RpoC interaction region	This study
	R‐rpoC: 5′‐TTGAGCTTGTCGACGGTCTG‐3′				
*ahp*C‐and its promoter	F‐ahpC: 5′‐CCGCAACGTCGACTGGCTCATA‐3′	62°C	835	*ahp*C promoter and full coding region	This study
	R‐ahpC: 5′‐TTCCCGGCCAACCAGATCCCGG‐3′				

*Note*: *rpo*B gene: N‐terminal region (codons 111–200): C‐I: Cluster I (codons 424–456); RRDR: Rifampicin Resistance Determining Region (codons 426–452); C‐II: Cluster II (codons 481–494); C‐III: Cluster III (codons 603–609); Quinolones resistance determining region (QRDR): *gyr*A (codons 74–133) and *gyr*B (codons 461–501).

PCR products were sequenced in both forward and reverse directions by Eurofins MWG Operon (Ebersberg, Germany). Each sequence was analysed and aligned with corresponding reference gene sequences of *M. tuberculosis* H37Rv strain (GenBank No. NC.000962.3) for the mutation identification using Bioedit software (version 7.1.10).

### Statistical analysis

The two‐tailed Fisher's exact test, odds ratio, and 95% confidence interval (95% CI) were performed to analyse the association of drug‐resistant associated mutations, levels of drug resistance, drug resistance groups, and compensatory mutations between *M. tuberculosis* genotypes. The difference with a *p*‐value <0.05 was considered to be statistically significant.

## RESULTS

### Drug resistance‐associated mutation profiles

**FIGURE 1:**
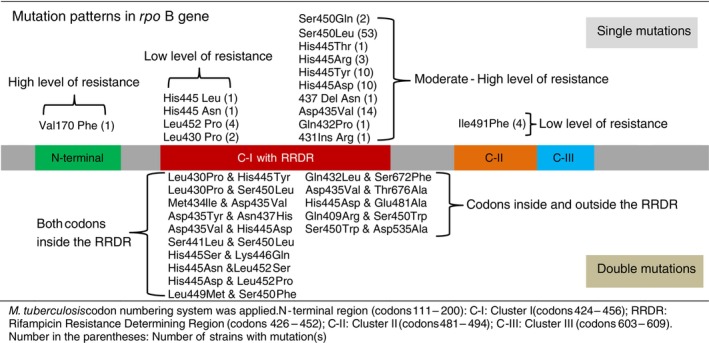
Mutations detected in the *rpo*B gene of the 126 rifampicin‐resistant strains.

Known rifampicin‐resistant mutations in the *rpo*B gene were found in 125 (99.2%) out of the 126 rifampicin‐resistant selected strains (Figure 1, Table 3, and Data S1). Thirty mutation patterns were detected, and mutations in the RRDR covered 95.2% of all rifampicin‐resistant strains. The mutation *rpo*B_Ser450Leu was the most common (*n* = 58, 46.4%), followed by *rpo*B_Asp435Val (12.7%), *rpo*B_His445Asp (11.9%) and *rpo*B_His445Tyr (7.9%) (Table 3). Only one and four rifampicin‐resistant strains carried the mutations *rpo*B_Val170Phe and *rpo*B_Ile491Phe, respectively, which are located in the N‐terminal and cluster‐II regions of the *rpo*B gene.

**TABLE 3:** Summary of major drug‐resistant and compensatory mutations in drug‐resistant strains across different *M. tuberculosis* lineages.

Gene/loci and major codon/nucleotide change(s) associated with resistance	Function of mutation	Number of drug‐resistant mutants	Proportion of mutations^a^ (%)	Proportion of mutations in *M. tuberculosis* lineages^b^
*rpo*B mutations	RIF resistance	125	99.2%	Beijing (77/77, 100%), EAI (30/30, 100%), EA (18/19, 94.7%)
*rpo*B mutations at codon 450		63	50%	Beijing (41/77, 53.2%), EAI (12/30, 40.0%), EA (10/19, 22.6%)
*rpo*B_Ser450Leu		58	46.4%	Beijing (40/77, 51.9%), EAI (10/30, 33.3%), EA (8/19, 42.1%)
*rpo*B mutations at codon 445		32	25.4%	Beijing (16/77, 20.8%), EAI (13/30, 43.3%), EA (3/19, 15.8%)
*rpo*B_His445Asp		15	11.9%	Beijing (7/77, 9.1%), EAI (7/30, 23.3%), EA (1/19, 5.3%)
*rpo*B_His445Tyr		9	7.9%	Beijing (5/77, 6.5%), EAI (3/30, 10%), EA (1/19, 5.3%)
*rpo*B mutations at codon 435		18	14.3%	Beijing (12/77, 15.6%), EAI (3/30, 10%), EA (3/19, 15.8%)
*rpo*B_Asp435Val		16	12.7%	Beijing (12/77, 15.6%), EAI (2/30, 6.7%), EA (2/19, 10.5%)
*rpo*C mutations	Compensatory mutations in RIF‐resistant strains	16	12.7%	Beijing (12/77, 15.6%), EAI (2/30, 6.7%), EA (2/19, 10.5%)
*rpo*A mutations		4	3.2%	Beijing (4/77, 5.2%)
Putative compensatory mutations in *rpo*B		5	3.9%	Beijing (2/77, 2.6%), EA (3/19, 15.8%)
*rpo*B_Ser450Leu mutants carrying *rpo*A‐*rpo*C		14	11.1%	Beijing (13/77, 16.9%), EAI (1/30, 3.3%)
Putative compensatory mutations in *rpo*A, *rpo*B and *rpo*C genes	Compensatory mutations in MDR strains	24	19.5%	Beijing (16/76, 21.1%), EAI (1/30, 3.3%), EA (7/17, 41.2%)
*kat*G mutations	INH resistance	146	85.9%	Beijing (94/100, 94.0%), EAI (36/45, 80.0%), EA (16/25, 64.0%)
*kat*G_Ser315Thr		130	76.5%	Beijing (91/100, 91.0%), EAI (27/45, 60.0%), EA (12/25, 48.0%)
Mutations in *inh*A and/or *inh*A promoter		30	17.6%	Beijing (10/100, 10.0%), EAI (15/45, 33.3%), EA (5/25, 20.0%)
*inh*A‐15 (C‐T)		27	15.9%	Beijing (9/100, 9.0%), EAI (13/45, 28.9%), EA (5/25, 20.0%)
*ahp*C mutations	Putative compensatory mutations in INH‐resistant strains	2	1.2%	EAI (1/45, 2.2%), EA (1/25, 4.0%)
*gyr*A‐*gyr*B mutations^c^	Fluoroquinolones resistance	25	20.3%	Beijing (14/76, 18.4%), EAI (6/30, 20.0%), EA (5/17, 29.4%)
*gyr*A_Asp94Gly^c^		11	8.9%	Beijing (6/76, 7.9%), EAI (2/30, 6.7%), EA (3/17, 17.6%)
*rrs*_1400 region mutation^c^	Resistance to second‐line injectable drugs	15	12.2%	Beijing 10/76, 13.2%), EAI (5/17, 29.4%)
*rrs*_1401 (A‐G)^c^		14	11.4%	Beijing (9/76, 11.8%), EAI (5/30, 16.7%)

^a^ Proportion: RIF‐resistant strains (*n* = 126), MDR (*n* = 123), INH‐resistant strains (*n* = 170).

^b^ Proportion in *M. tuberculosis* lineages: lineage 1/East African–Indian (EAI) genotypes (total strains = 45, MDR = 30), lineage 2/Beijing genotypes (total strains = 101, MDR = 76), lineage 4/Euro‐American (EA) genotypes (H, LAM, T and U) (total strains = 27, MDR = 17).

^c^ Proportion of mutations was calculated for MDR strains (*n* = 123).

**FIGURE 2:**
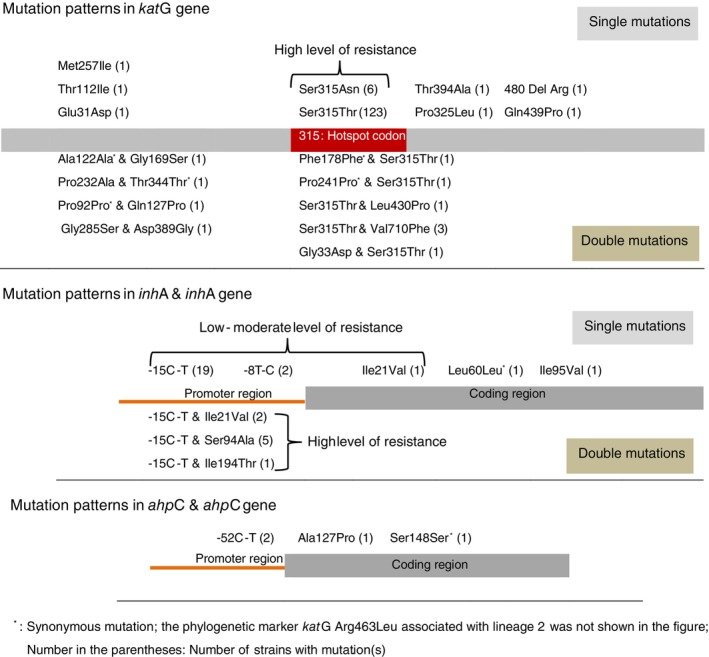
Mutations detected in the *kat*G, *inh*A, *inh*A promoter, *ahp*C, and *ahp*C promoter of the 170 isoniazid‐resistant strains.

Isoniazid‐resistant mutations were mainly found in *kat*G (*n* = 146, 85.9%), followed by *inh*A and/or *inh*A promoter (*n* = 30, 17.6%) of isoniazid‐resistant strains (Figure 2a, b, Table 3 and Data S1). 11 isoniazid‐resistant strains carried mutations in both genes. Combinations of *kat*G and *inh*A mutations covered up 97.1% (165/170) of isoniazid‐resistant strains, while only *kat*G_Ser315Thr (*n* = 130, 76.5%) and *inh*A‐15(C‐T) (*n* = 27, 15.9%) accounted for 93.5%.

A total of 25 out of 123 (20.5%) MDR strains carried fluoroquinolone‐resistant mutations in either *gyr*A (*n* = 23) or *gyr*B (*n* = 2) (Table 3 and Data S1). The most frequent mutations in *gyr*A were *gyr*A_Asp94Gly (11/23, 47.8%), followed by *gyr*A‐Asp90Val (6/23, 26.1%). Overall, the mutations in *gyr*A at codon 94 (*gyr*A_Asp94Ala, *gyr*A_Asp94Gly, *gyr*A_Asp94Gln and *gyr*A_Asp94Tyr) associated with *high‐level* fluoroquinolone *resistance* accounted for 69.6% (16/23) of the fluoroquinolone‐resistant strains. Second‐line injectable drug resistance mutations were found in 15 out of 123 (12.2%) MDR strains, and most of these mutations displayed at position *rrs*_1401A‐G (14/15, 93.3%) (Table 3 and Data S1), which is known to correlate with high‐level resistance to kanamycin, amikacin, and capreomycin and carries low fitness cost.

### Compensatory mutations in RIF and INH resistant strains

**FIGURE 3:**
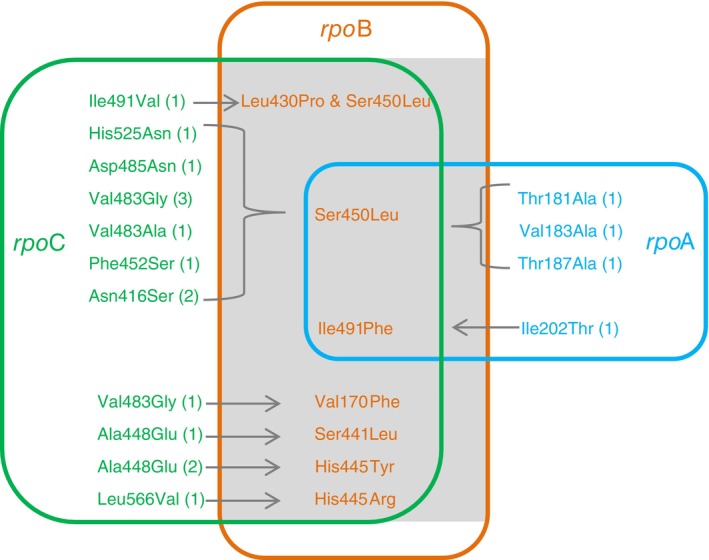
Putative compensatory mutations in *rpo*A and *rpo*C genes associated with *rpo*B mutations in clinical MDR *M. tuberculosis* strains. Number in the parentheses: Number of strains with mutation(s).

Thirty‐five rifampicin‐resistant strains revealed a secondary mutation within one of the genes *rpo*A (*n* = 4), *rpo*B (*n* = 15) and *rpo*C (*n* = 16) (Table 3 and Figure 3). The compensatory mutations in *rpo*A and *rpo*C genes were mainly found in RIF‐resistant strains carrying mutations in the RRDR, but were also detected in strains with mutations in the Cluster II and N‐terminal region of the *rpo*B gene. The proportion of *rpo*A‐*rpo*C mutants harbouring the mutation *rpo*B_Ser450Leu (14/20, 70.0%) was statistically significantly higher than those with other *rpo*B mutations (OR = 5.4; 95% CI = 1.4 to 21.1, *p* = 0.02). The RIF‐resistant strains carrying the mutations *rpo*B_Val170Phe and *rpo*B_Ile491Phe also carried compensatory mutations in *rpo*A‐*rpo*C genes.

Fifteen double *rpo*B mutation patterns were observed (Figure 1 and Data S1), among which 10 strains showed secondary mutations known to also be RIF‐resistance mutations, and five others revealed secondary mutations, *rpo*B_Gln409Arg, *rpo*B_Glu481Ala, *rpo*B_Asp535Ala, *rpo*B_Ser672Phe, and *rpo*B_Thr676Ala, located outside the RRDR region of the *rpo*B gene not associated with rifampicin resistance.

Only two out of the 170 isoniazid‐resistant strains harboured the mutation at position −52(C‐T) in the promoter region of the *ahp*C gene (Figure 2c). Nevertheless, the *ahp*C‐52(C‐T) mutation was found in one isolate carrying a mutation in *kat*G Pro325Leu, while the other isolate was wild‐type for *kat*G and *inh*A.

### Association of drug‐resistant mutations and compensatory mutations with *M. tuberculosis* genotypes

The *rpo*B mutations at codons 450 and 435 were more frequently found in Beijing and “other genotype group” (68.8% and 68.4%, respectively) than in EAI genotypes (50%) (OR = 2.2, 95% CI = 0.9 to 5.2, *p* = 0.07). Similarly, the proportion of *rpo*B_Ser450Leu and *rpo*B_Asp435Val mutations was higher in the Beijing and EA genotypes than in the EAI genotypes (Table 3). Nevertheless, the frequency of *rpo*B mutations at codon 445 was significantly higher in EAI genotypes compared to Beijing genotypes (43.3% vs. 20.8%, OR = 2.9; 95% CI = 1.2–7.2, *p* = 0.02), while the difference between EAI and EA genotypes (15.8%) was not statistically significant (OR = 4.1; 95% CI = 1.0–17.0, *p* = 0.06).

The compensatory mutations in *rpo*A, *rpo*B, and *rpo*C were found in 19.5% (24/123) of MDR strains (Table 3). The frequency of compensatory mutations in *rpo*A, *rpo*B, and *rpo*C in Beijing genotypes was significantly higher than in the EAI genotype (21.1% vs. 3.3%, OR = 7.7, 95% CI = 1.0 to 61.2, *p* = 0.03) A similar trend was observed for EA genotypes, which exhibited a significantly higher compensatory mutation frequency than EAI genotypes (41.2% vs. 3.3%, OR = 20.3; 95% CI = 2.2–186.0, *p* = 0.002). The combination of the rifampicin‐resistant mutation *rpo*B_Ser450Leu and the compensatory mutations in the *rpo*A, *rpo*B, and *rpo*C genes was strongly associated with Beijing genotypes more than with all other genotypes in this sample set (OR = 9.9, 95% CI = 1.2 to 78.4, *p* = 0.001).

Among the 130 isoniazid‐resistant strains carrying the mutation *kat*G_Ser315Thr, the frequencies significantly differed among Beijing genotypes (93/100, 93.0%) and EAI genotypes (60.0%) (OR = 6.7; 95% CI = 2.7 to 16.7, *p* = 0.00002), and EA genotypes (48.0%) (OR = 10.9; 95% CI = 3.9 to 31.0, *p* = 0.000006) (Table 3). On the contrary, the proportion of *inh*A‐15(C‐T) mutation in EAI genotypes (28.9%) was significantly higher than in Beijing genotypes (9.0%) (OR = 4.1; 95% CI = 1.6 to 10.5, *p* = 0.003), but the difference was not significant compared with EA genotypes (20%) (OR = 1.6; 95% CI = 0.5 to 5.3, *p* = 0.57). The putative compensatory mutation of isoniazid resistance *ahp*C‐52(C‐T) in the promoter region of the *ahp*C gene was found in EAI and EA genotypes.

## DISCUSSION

### Drug‐resistance and compensatory mutations

It is well known that drug‐resistance mutations are often accompanied by a fitness cost, consequently leading to less competitive drug‐resistant strains compared to drug‐sensitive counterparts [2, 3, 12]. Nevertheless, the acquisition of mutations associated with moderate to high levels of phenotypic drug resistance without or with low fitness cost seems an effective evolutionary pathway in *M. tuberculosis* [9, 14, 16, 26]. In our analysis, mutations in antibiotic resistance‐associated genes previously shown to confer low‐to‐high levels of phenotypic drug resistance with low or no fitness cost compared to the wild type, including *rpo*B_Ser450Leu, *kat*G_Ser315Thr, *inh*A‐15(C‐T), *gyr*A_Asp94Gly, and *rrs*_1401(A‐G) were commonly detected at a high proportion (46.3%, 76.4%, 16.2%, 8.9%, and 11.4%, respectively) in MDR *M. tuberculosis* strains. These mutations allow *M. tuberculosis* strains to thrive despite the selective pressure of the treatment, often leading to the persistence or spread of resistant strains in the population [16, 17, 18, 19, 26, 27, 28, 29]. Recently, the WHO published mutation catalogues for *M. tuberculosis* complex, detailing their association with phenotypic drug resistance (first edition in 2021, second edition in 2023) to promote the adoption of molecular diagnostics by national tuberculosis programmes [30, 31, 32]. Our findings provide valuable data that could contribute to these catalogues, enhancing the interpretation of genome sequencing results and informing the development of new molecular drug susceptibility tests, including targeted next‐generation sequencing.

In addition, we found that 19.5% of MDR t strains carried at least one compensatory mutation in either the *rpo*A, *rpo*B, or *rpo*C gene, which is in concordance with global studies [10, 14, 18, 19, 22, 23]. The mutations in *rpo*A and *rpo*C clustered in regions of the RNA polymerase encoding the *interface* of the *RNA polymerase* subunits (the RpoA‐RpoC interface), and secondary mutations located outside the RRDR of the *rpo*B gene could alleviate the biological cost of rifampicin‐resistant *rpo*B mutations [15, 26]. Recent whole‐genome sequencing studies of MDR *M. tuberculosis* strains have revealed that putative compensatory mutations of rifampicin resistance are dispersed across a broader range in the *rpo*A, *rpo*B, and *rpo*C genes than previously recognised [33, 34, 35, 36, 37]. Although compensatory mutations play a critical role in mitigating the fitness costs associated with rifampicin resistance, thereby enhancing the survival and transmission of drug‐resistant strains [14, 26, 33, 35, 36, 38], these mutations are not only contributors to the current epidemic of MDR‐TB [34, 39]. Holt et al. 2018 identified a potential mutation EsxW‐Thr2Ala (an ESX‐5 type VII secreted protein) promoting the enhanced transmission of Beijing lineage strains in Vietnamese and other host populations [39]. Therefore, along with compensatory mutations of antibiotic resistance, other mutations could contribute to the transmission dynamics of the *M. tuberculosis* population at different levels. Our study focused specifically on sequencing well‐characterised target genetic regions in the *rpo*A, *rpo*B, and *rpo*C genes associated with RIF resistance and known compensatory mutations. This targeted approach may have overlooked compensatory mutations located outside the predefined regions. Future studies incorporating comprehensive whole‐genome analyses could provide a more complete understanding of the current trend of transmission of MDR/XDR *M. tuberculosis* strains in Vietnam. Finally, strengthening surveillance and monitoring efforts is essential to curb the rapid spread of highly transmissible clones that can quickly acquire resistance mutations to newly approved drugs such as bedaquiline, delamanid, and pretomanid.

In the case of isoniazid resistance, it was largely demonstrated that the *kat*G_Ser315Thr mutation has low or no fitness cost [27] explaining why this mutation has been favoured in drug‐resistant strains worldwide [5]. Although none‐*kat*G315_Ser315Thr mutations associated with isoniazid resistance are determined to be biologically costly mutations, the compensatory mutation in the *ahp*C promoter is globally rare [27]. Accordingly, we found only 1.2% of isoniazid‐resistant isolates carrying the *ahp*C‐52(C‐T) promoter mutation, which has never been reported before. For the *inh*A gene, the no‐cost *inh*A‐15(C‐T) promoter mutation was the most common, which leads to isoniazid resistance through the overexpression of InhA protein [2]. In this study, mutations in the *inh*A promoter were either alone or accompanied by secondary mutations (also known to be associated with isoniazid resistance) in either the *kat*G or *inh*A gene. Although the accumulation of mutations in the *inh*A and *ahp*C genes resulted in high levels of isoniazid resistance among non‐*kat*G315_Ser315Thr mutations in MDR *M. tuberculosis* isolates, these combinatorial mutations were less frequently observed [40]. In agreement with previous studies [7, 16, 40, 41], our findings provided insights into the correlation between combinatorial mutations with levels of drug resistance and transmission of drug‐resistant *M. tuberculosis* in the population which would aid in developing better strategies for the diagnosis and management of MDR‐TB.

### The association between drug‐resistance mutations, compensatory mutations, and the genetic background of *M. tuberculosis*

**FIGURE 4:**
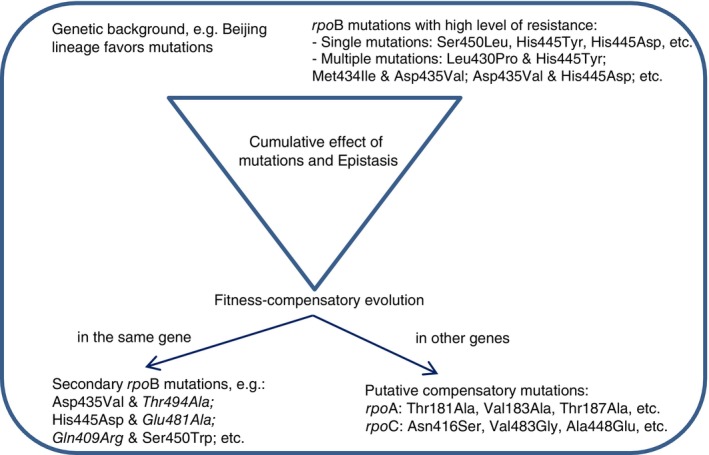
The association between intrinsic factors: Genetic background, drug resistance‐associated mutations, and compensatory mutations in the *M. tuberculosis* population in Vietnam.

It has been demonstrated that drug resistance and fitness‐compensatory evolution vary according to the *M. tuberculosis* lineages [3, 42, 43, 44]. Here, we found strong interactions between intrinsic factors including drug resistance‐associated mutations, fitness‐compensatory mutations, and genetic background (Figure 4), suggesting that the cumulative effect of mutations and epistasis could drive the evolution of drug resistance and transmission of *M. tuberculosis* in Vietnam. Specifically, among the isoniazid‐resistant strains, we found a significantly higher proportion of *inh*A‐15(C‐T) mutation in lineage 1/EAI genotypes compared to lineage 2/Beijing genotypes, while lineage 1 has a significantly lower proportion of *kat*G315_Ser315Thr mutations compared to lineage 2 and lineage 4/EA genotypes. In the context of rifampicin resistance, Beijing genotypes exhibited a higher prevalence of *rpo*B mutations at codons 450 and 445 compared to AE genotypes and at codons 450 and 435 compared to EAI genotypes. Notably, EAI genotypes showed a significantly higher frequency of mutations at codon 445 than Beijing genotypes, highlighting distinct mutational patterns across *M. tuberculosis* lineages.

Overall, Beijing genotypes were mainly linked to mutations associated with highlevels of phenotypic drug resistance, while EAI genotype was often associated with mutations conferring low – moderate levels of phenotypic drug resistance. In agreement with previous findings [5, 16, 18, 22, 23, 28], the high prevalence of mutations at codons *kat*G_Ser315Thr and *rpo*B_Ser450Leu in the Beijing strains demonstrates the evidence that this genotype is associated with MDR phenotypes and has recently been widely spread in the world. Regarding the compensatory mutations, we also found a strong association between mutations in *rpo*B and *rpo*A – *rpo*C with Beijing genotypes, consistent with other studies [14, 16, 19]. Nevertheless, in our sample, the same compensatory mutations were found in different genotypes, suggesting independent acquisition. Specifically, *M. tuberculosis* strains of EAI genotypes revealed mutations in only the *rpo*C gene; strains of EA genotypes carried more specific secondary mutations in the *rpo*B gene, while strains of Beijing genotypes tended to accumulate secondary mutations in *rpo*A –*rpo*C genes but also in the *rpo*B gene. This finding is in concordance with previous studies in which the proportion of compensatory mutations in rifampicin‐resistant strains was found to be 30% to 86% [10, 19] *and significantly associated with rpoB_Ser450Leu and* the *Beijing genotype* [16, 18, 41]. Thus, the multiple drug‐resistant Beijing strains successfully acquired multiple mutations associated with high levels of drug resistance, maintaining the high transmissibility capacity of this family. This multiple‐mutant genotype generally is the result of a sequential cumulative acquisition of mutations, and epistasis probably drives the fixation of mutation patterns [13, 28]. Since Beijing genotypes are responsible for more than a quarter of the TB prevalence worldwide [41], these data suggest an unfavourable scenario for the emergence and transmission of highly drug‐resistant strains, with a selective advantage.

Presently, various large‐scale phylogenomic analyses have provided insights into the complex processes of *M. tuberculosis* transmission underlying the evolutionary dynamics of this pathogen according to genetic background, geographical area, and demographics. Notably, Holt et al. investigated the transmission of *M. tuberculosis* strains in the South of Vietnam and found higher levels of transmission of Beijing lineage strains within this host population than endemic lineage 1 [39]. Specifically, Beijing sub‐lineage 2.2.1 exhibited significantly high proportions of mutations associated with resistance to rifampicin, isoniazid, streptomycin, and ethambutol compared to all non‐sub‐lineage 2.2.1. Consistently, major mutations such as *rpo*B_Ser450Leu and *emb*B_Met306Val were more frequent in Beijing sub‐lineage 2.2.1 than in other lineages and sub‐lineages. Notably, Beijing sub‐lineage 2.2.1 strains exhibited a significantly shorter evolutionary time of transmission than Beijing sub‐lineage 2.2.2 and non‐Beijing lineage strains. In addition, this study provided evidence that *M. tuberculosis* sub‐lineage 1.1.1.1 is endemic to Vietnam, with its transmission predominantly confined to this region and a few neighbouring areas. The limited geographical spread of this sub‐lineage suggests that it has become highly adapted to the local host population, with which it has likely co‐evolved over centuries. This pattern of restricted transmission and host adaptation mirrors the behaviour of host‐specialist clades recently identified within *M. tuberculosis* Lineage 4 [44], further supporting the concept of geographic and host‐specific evolution in this pathogen. This finding highlights the significant role of the genetic background of *M. tuberculosis* lineages and sub‐lineages in shaping the evolutionary pathways of antibiotic resistance in this pathogen.

A limitation of this study lies in the potential selection bias of the *M. tuberculosis* strains included in our sample. These strains were collected in 2009, and as such, this temporal gap suggests that the proportions of drug‐resistant strains in our dataset may not accurately reflect the up‐to‐date epidemiological trends of drug‐resistant *M. tuberculosis* in Vietnam. Despite this limitation, the findings provide valuable insights into the compensatory mutations in MDR *M. tuberculosis* strains circulating in Vietnam at the time. Understanding these mechanisms of compensatory mutation evolution and their role in the transmission dynamics of MDR strains is crucial for anticipating how drug resistance may evolve in the country, thereby contributing to more effective strategies for controlling drug‐resistant tuberculosis in Vietnam.

## CONCLUSION

Our study gave more insights into genetic determinants associated with drug resistance and compensatory mutations of Beijing, EAI, and other *M. tuberculosis* genotypes in Vietnam. These findings underline that a genomic‐based surveillance is necessary to bring valuable insights for drug resistance and transmission control in *M. tuberculosis*. Given that the tuberculosis incidence has decreased in Vietnam from 2007 to 2017 by 3% each year [21], the incidence of MDR/RR TB has increased from 7600 cases to 9200 cases between 2015 and 2022 [1], it would be highly relevant to explore the evolution over time of the genetic determinants and compensatory mutations of the *M. tuberculosis* population circulating in Vietnam to date, using next‐generation sequencing methodologies. These data could explain why the proportion of MDR strains is increasing although the global incidence is decreasing and why overall incidence is rising again.

## AUTHOR CONTRIBUTIONS

Conception and design: Quang Huy Nguyen, Anne‐Laure Bañuls; Data analysis and interpretation: Quang Huy Nguyen, Thi Van Anh Nguyen, Anne‐Laure Bañuls; Manuscript writing: All authors; Final approval of manuscript: All authors.

## CONFLICT OF INTEREST STATEMENT

The authors declare no conflict of interest.

## Supporting information


**DATA S1.** Supporting Information.
